# An unusual pancreas divisum intraductal papillary mucinous neoplasm diagnosed by pancreatoscopy-assisted endoscopic ultrasonography rendezvous procedure

**DOI:** 10.1055/a-2261-7642

**Published:** 2024-02-22

**Authors:** Belén Agudo Castillo, Daniel de la Iglesia, Carlos Esteban Fernández-Zarza, Esther Maderuelo González, Ana Isabel Cabrero Cabrero, Paula Aguirre-Camino, Mariano González-Haba Ruiz

**Affiliations:** 1 Department of Gastroenterology, Puerta de Hierro University Hospital, Majadahonda, Spain; 2 Department of Radiology, Puerta de Hierro University Hospital, Majadahonda, Spain

This video case report presents the endoscopic management of a main duct intraductal papillary mucinous neoplasm (IPMN) in a 75-year-old patient with a history of acute pancreatitis.

The patient presented at the emergency department with an episode of acute pancreatitis. Based on endoscopic ultrasound (EUS) and magnetic resonance imaging (MRI), a main duct IPMN was suspected with a pancreas divisum. A pancreatoscopy to establish the extension of the main duct IPMN was planned prior to surgery ( Video 1 ) 1 2 . Pancreatoscopy through the major papilla revealed typical features of IPMN, such as mucinous secretion and papillary mucosal changes. However, due to the anatomical complexity associated with pancreas divisum, a complete examination of the dorsal pancreas was not feasible through this approach. To overcome the limitations posed by pancreas divisum and the impossibility to identify the minor papilla during endoscopic retrograde cholangiopancreatography (ERCP) 3 , a rendezvous procedure was undertaken ( Fig. 1 ). Utilizing EUS guidance, a guidewire was passed through the minor papilla and through the use of this guidewire, the pancreatoscope was advanced to the dorsal pancreas via the minor papilla. This innovative approach provided a comprehensive view of the pancreatic ductal system.

Endoscopic evaluation of a main duct intraductal papillary mucinous neoplasm in a patient with pancreas divisum.Video 1

**Fig. 1:**
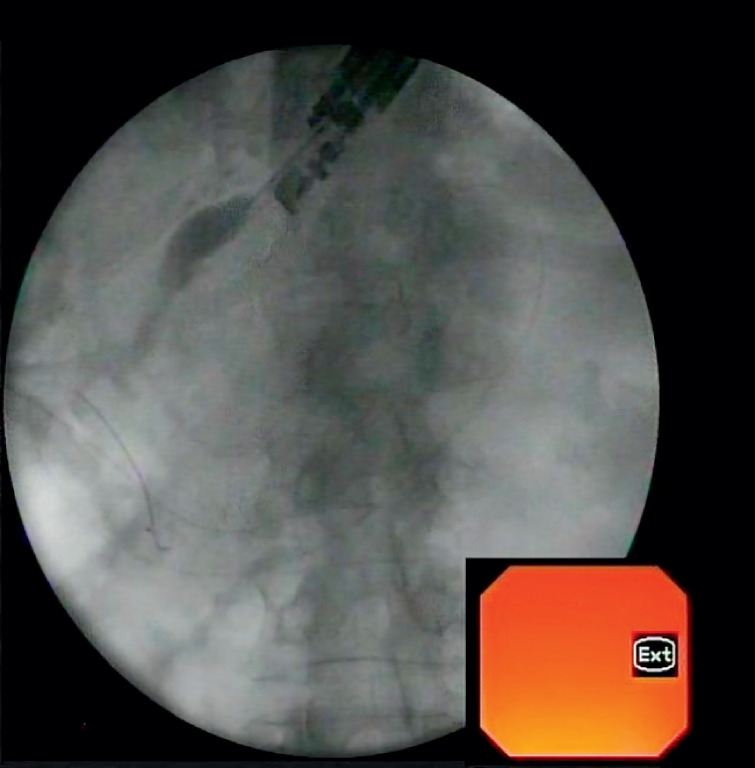
Endoscopic ultrasound-guided rendezvous technique.

The video demonstrates a normal dorsal pancreas and Santorini duct, confirming the absence of pathological changes in these regions. This comprehensive examination led to a final diagnosis of ventral duct IPMN, with no involvement of the dorsal pancreatic duct. The video highlights the importance of a thorough and tailored endoscopic approach in patients with complex pancreatic anatomy like pancreas divisum.

Endoscopy_UCTN_Code_TTT_1AS_2AD

## Notes

Endoscopy E-Videos https://eref.thieme.de/e-videos
*E-Videos* is an open access online section of the journal *Endoscopy* , reporting on interesting cases and new techniques in gastroenterological endoscopy. All papers include a high-quality video and are published with a Creative Commons CC-BY license. Endoscopy E-Videos qualify for HINARI discounts and waivers and eligibility is automatically checked during the submission process. We grant 100% waivers to articles whose corresponding authors are based in Group A countries and 50% waivers to those who are based in Group B countries as classified by Research4Life (see: https://www.research4life.org/access/eligibility/ ). This section has its own submission website at https://mc.manuscriptcentral.com/e-videos .
